# Supplementary material for: Latent ion tracks were finally observed in diamond
Source: Nat Commun. 2024 Feb 27;15:1786. doi: 10.1038/s41467-024-45934-4 (PMC10899563; doi:10.1038/s41467-024-45934-4)
Supplement: Supplementary file 1 — Supplementary Information [file 41467_2024_45934_MOESM1_ESM.pdf]

Supplementary Information

Latent ion tracks were finally observed in diamond

H. Amekura[1]*, A. Chettah[2], K. Narumi[3], A. Chiba [3], Y. Hirano[3], K. Yamada[3], S. Yamamoto[3], A.A. Leino[4], F. Djurabekova[4], K. Nordlund[4], N. Ishikawa[5], N. Okubo[5] & Y. Saitoh[3]

[1] National Institute for Materials Science (NIMS), Tsukuba, Ibaraki 305-0003, Japan.
[2] Department of Physics, LGMM laboratory, University of 20 Août 1955-Skikda, BP 26, route d'El Hadaiek-Skikda 21000-Algeria.
[3] Takasaki Institute for Advanced Quantum Science, National Institutes for Quantum Science and Technology (QST), Takasaki 370-1292, Japan.
[4] Department of Physics and Helsinki Institute of Physics, University of Helsinki, PO Box 43, FI-00014 Helsinki, Finland.
[5] Japan Atomic Energy Agency (JAEA), Tokai, Ibaraki 319-1195, Japan.

Table of Contents

Supplementary Note 1. Confirmation of the ion tracks by the over-/under- defocusing

Supplementary Note 2. Ellipsoidal tracks by the inclined ion incidence of 7°

Supplementary Note 3. The determination of the track diameters from TEM side-view images

Supplementary Note 4. The high-angle annular dark-field (HAADF) STEM image of the tracks

Supplementary Note 5. Two temperature molecular dynamics simulations

Supplementary Note 6. The highest electronic stopping power induced by monoatomic ions in diamond

Supplementary Note 7. Almost one grain structures of microscopically thinned samples for TEM observation

Supplementary Note 8. Purity of C_{60} cluster ion beam

Supplementary Note 9. Validity of the independent sixty C ion model for estimating the stopping power of C_{60} ion

Supplementary Methods 1: Two configurations of TEM sample fabrication (Top-view/ Side-view)

Supplementary Note 1. Confirmation of the ion tracks by the over-/under- defocusing

Supplementary Figure 1 shows bright-field TEM images of diamond irradiated with 6 MeV C_{60}^{+} ions to a fluence of 5×10^{10} C_{60} cm^{-2}. The nearly same area was observed at three different conditions of (a) over-focus, (b) In-focus, and (c) under-focus. In order to stress the changes in images, the absolute values of the defocus in (a) and (c) were higher than those used for other observations. Tracks observed as black dots in (a) the over-focus condition turned to white dots in (c) the under-focus condition. The contrast reversal with defocus condition indicates that the dots were observed as the Fresnel-contrast [1,2]. Therefore, the contrast reversal is evidence that the observed dots have lower density, i.e., ion tracks.

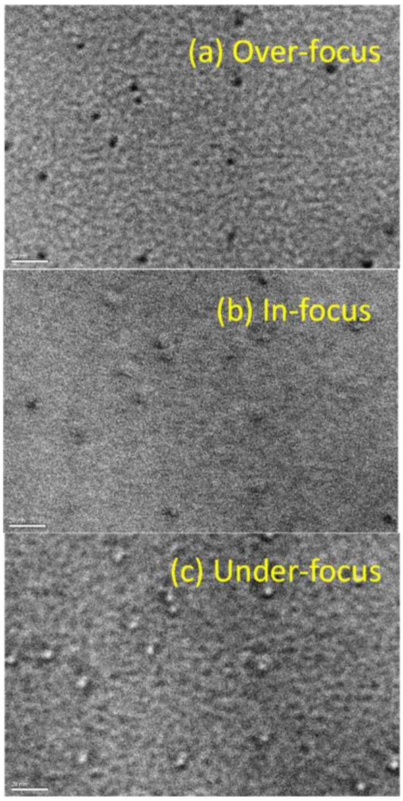

Supplementary Figure 1. Bright-field TEM images of top-views of diamond irradiated with 6 MeV C_{60}^{+} ions to a fluence of 5×10^{10} C_{60} cm^{-2}. The nearly same area was observed at three different conditions of (a) over-focus, (b) in-focus, and (c) under-focus. Tracks observed as black dots in (a) the over-focus condition turned to white dots at (c) under-focus condition. The scale bars are all 20 nm.

Supplementary Note 2. Ellipsoidal tracks by the inclined ion incidence of 7°

As shown in **Figure 2a in the main text**, the tracks observed from the surface normal direction are not perfectly circles but ellipses. This is probably due to the ion incident angle being 7° from the surface normal since the images of the track sidewalls overlapped with the track diameters as shown in **Supplementary Figure 2**. The contribution is roughly $L \cdot \sin 7° \sim 6$ nm, which is comparable to the track diameter, where L ~50 nm denotes the mean track length of 9 MeV C_{60} ions in diamond (See **Figure 3c in the main text**).

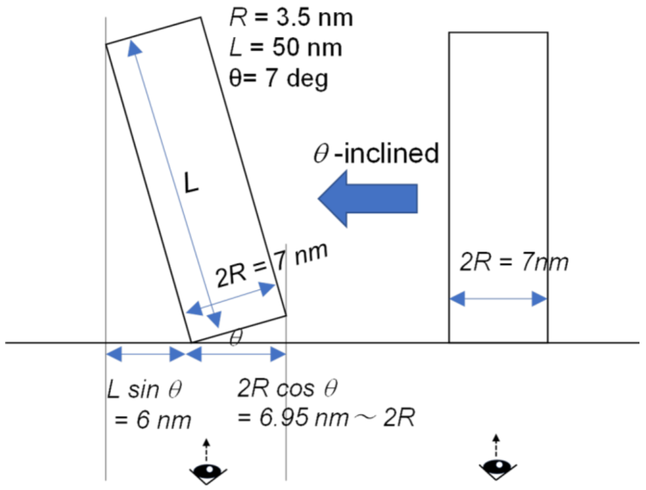

Supplementary Figure 2. A geometrical relationship of (elliptical) track width and the incident angle. The sidewall of the track contributes.

Supplementary Note 3. The determination of the track diameters from TEM side-view images

We used a very primitive method to set the boundary of a track. See **Supplementary Figure 3.** At first, the contrast of the image was optimized. (We confirmed this procedure does not the track diameter largely.) After then, a track was sandwiched by two parallel lines and the diameter was determined, as shown in **Supplementary Figure 3.** In the case of the tracks whose diameter changes with the depth, a kind of averaging via human's eye was done, and "averaged diameter along the depth" was determined.

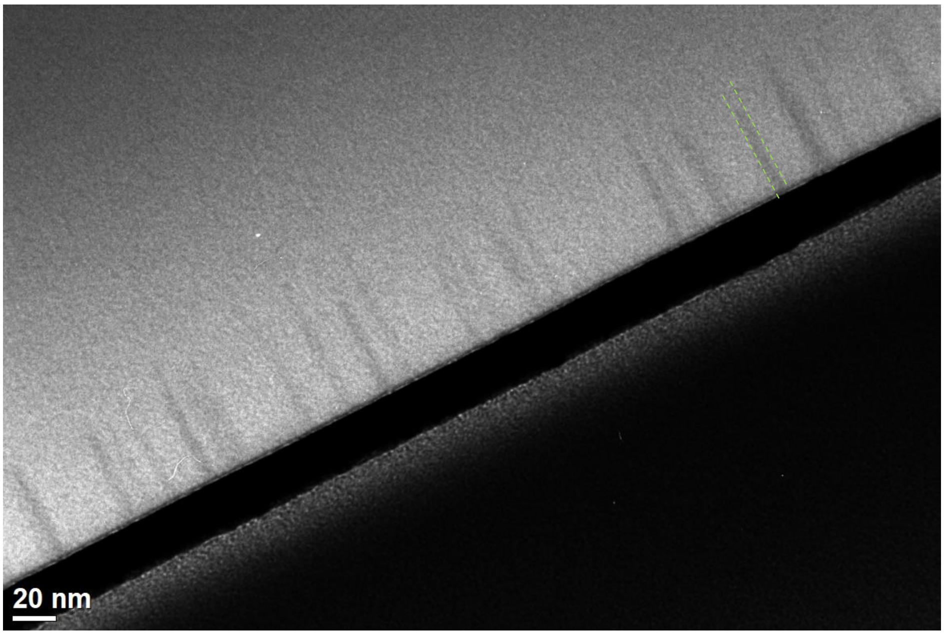

Supplementary Figure 3. A side-view BF-TEM image of diamond sample irradiated with 9 MeV C_{60} ions. The contrast was modified to clarify the track images. A pair of lines in the right indicates how to determine the track diameter.

Supplementary Figures 4a-d show BF-TEM side-view images of diamond samples irradiated with C_{60} ions of (a) 2 MeV, (b) 4 MeV, (c) 6 MeV, and (d) 9 MeV. Particular in the samples irradiated with 2 MeV ions, some tracks look not perfectly cylindrical. However, we have determined the diameters assuming the perfect cylinders, and then the determined mean diameter and SD were shown in **Figure 3c in the main text.** The SD is simply due to the statistical scattering of the diameters. The uncertainty due to the non-perfect cylinder shapes is not considered.

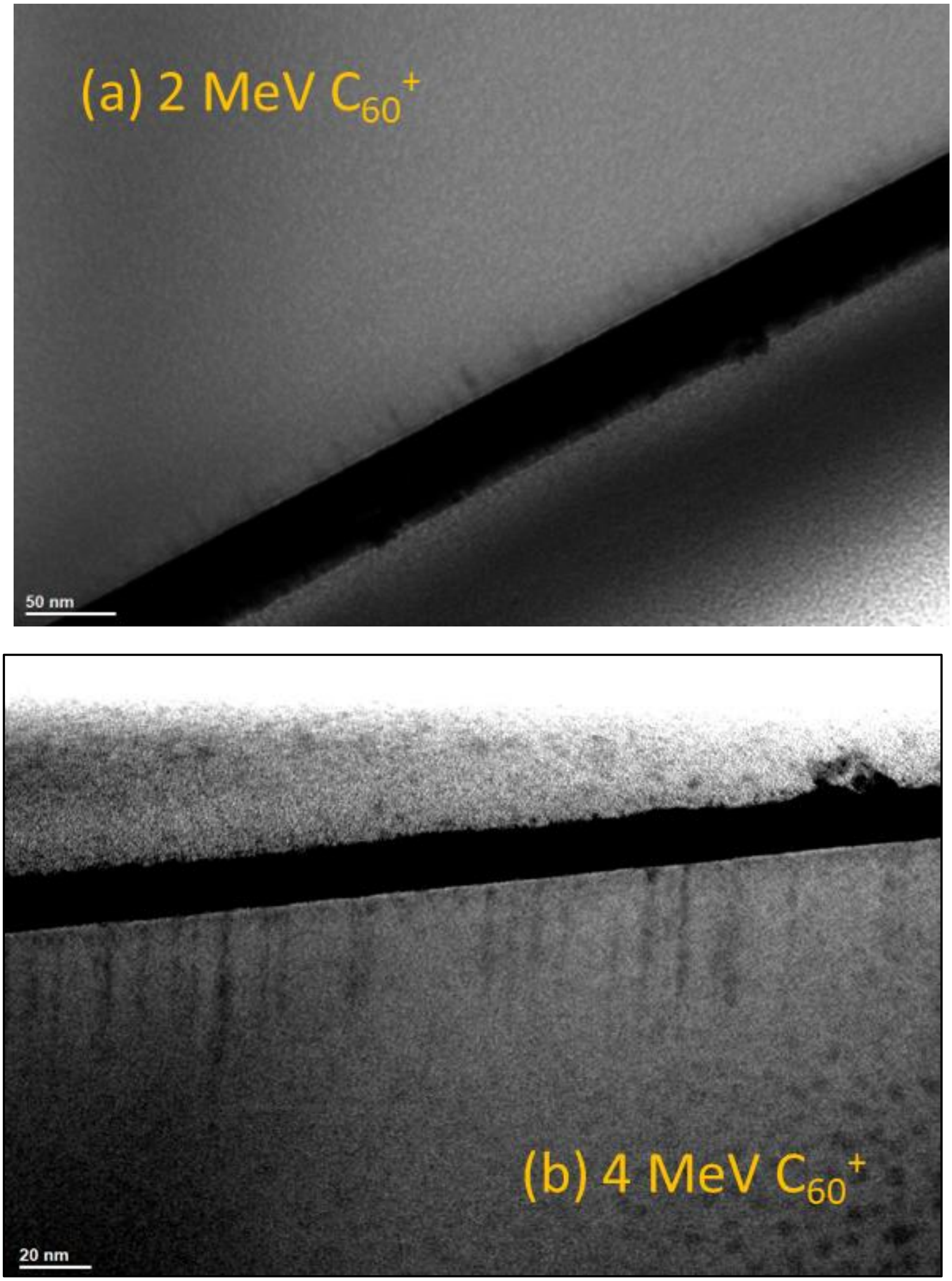

Supplementary Figure 4. BF-TEM side-view images of diamond samples irradiated with C_{60} ions of (a) 2 MeV, (b) 4 MeV, (c) 6 MeV, and (d) 9 MeV. The contrast was modified to clarify the ion track images.

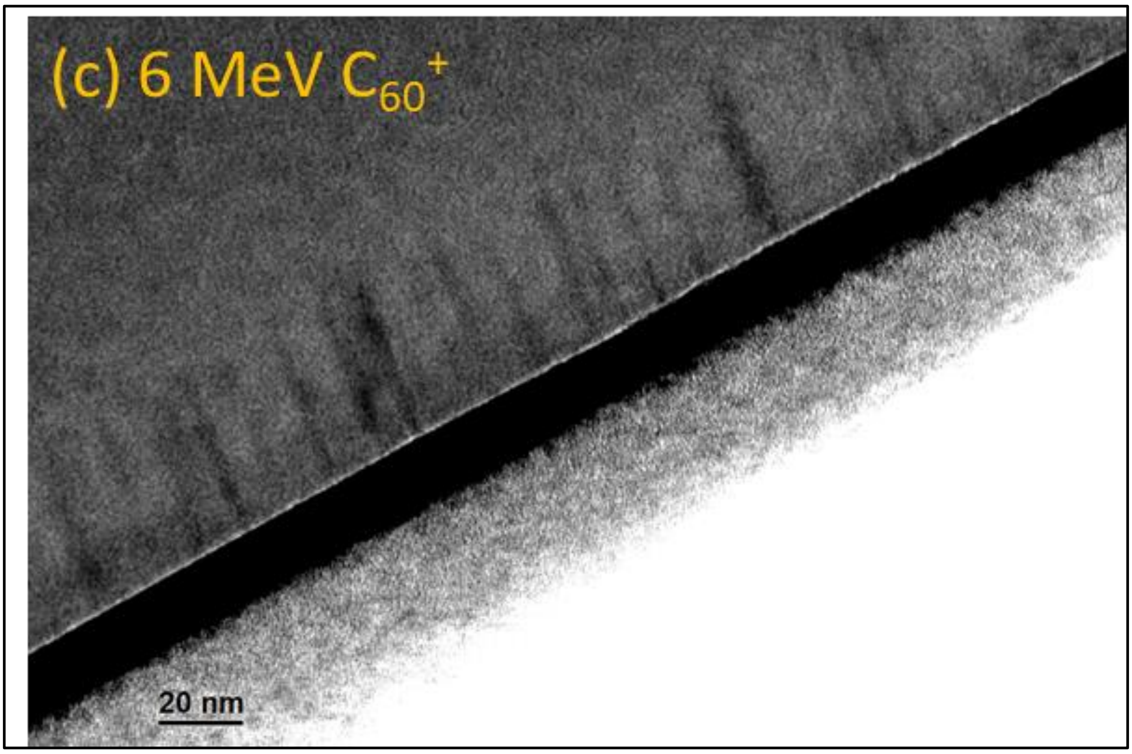
(c) 6 MeV C_{60}^{+}
20 nm

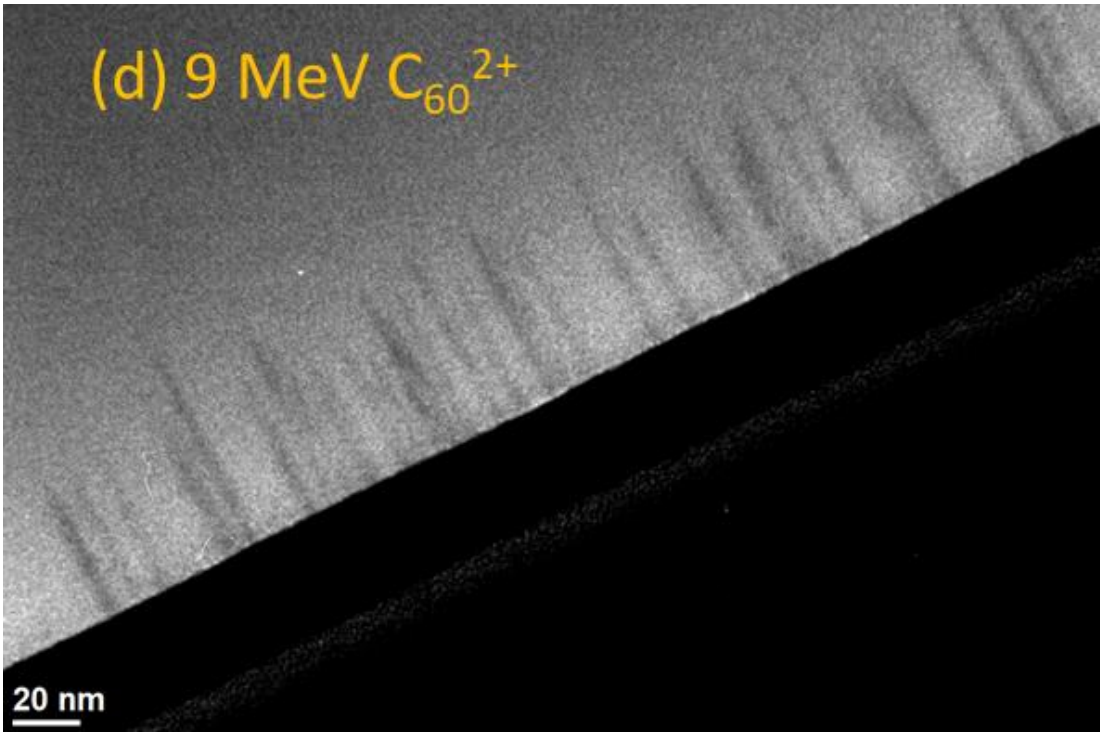
(d) 9 MeV C_{60}^{2+}
20 nm

Supplementary Note 4. The high-angle annular dark-field (HAADF) STEM image of the tracks

Supplementary Figure 5 shows a low-resolution HAADF image of a track in diamond irradiated with 9 MeV C_{60} ions. JEOL JEM-2100F was utilized with a beam spot of 0.5 nm, which does not have a spherical aberration correction. **Supplementary Figure 5** shows a black track core indicating a lower density or lesser thickness, which is surrounded by a slightly whity ring indicating a higher density or greater thickness.

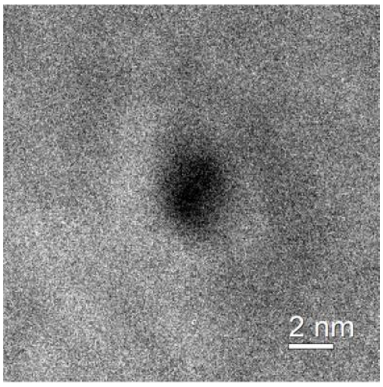

Supplementary Figure 5. A HAADF image of the ion track formed in diamond irradiated with 9 MeV C_{60} ions, observed by JEM-2100F with a beam spot of 0.5 nm, without the spherical aberration correction.

Supplementary Note 5. Tow temperature molecular dynamics simulations

Simulations using the two-temperature molecular dynamics model (TTMD) are performed following the method by Ivanov and Zhigilei [3], which is implemented in *PARCAS* MD code [4][5,6] and described in Ref. [7]. It incorporates electronic effects into classical molecular dynamics with a friction term

$$F_i = -\nabla_i V(\{\vec{r}\}) + \xi v_i \qquad (1)$$

where F_i is the force acting on the i:th particle, V is the Tersoff potential [8] and v_i the velocity. The second term provides coupling with the electronic part of the two-temperature

model [3],

$$C_e(T_e)\,\frac{\partial T_e}{\partial t} = \nabla(K_e(T_e)\cdot\nabla T_e) + G(T_e)(T_e - T_l), \qquad (2)$$

$$T_e(t=0) = A(r_\perp) \qquad (3)$$

which is solved on a 51 × 51 × 1 finite difference grid over the MD simulation domain (23 nm × 23 nm × 13 nm) in size. T_e is the electronic temperature, $C(T_e)$ the electronic heat capacity and $K_e(T_e)$ the electronic heat conductivity, written as $K_e(T_e) = C_e(T_e)D_e$, where D_e is the electronic heat diffusivity. $G(T_e)$ is the effective electron-phonon coupling and $A(r_\perp)$ describes the electronic temperature after the initial electronic collision cascade. Each grid cell has the same volume V_{cell}. Within the cell, local lattice temperature T_l is determined using the kinetic energy. The lattice temperature can be used to resolve the magnitude of the friction term ξ by requiring that the total energy of the system (heat equation + kinetic energy in MD simulation) is conserved at each timestep [3],

$$\xi = \frac{V_{cell}G(T_e)(T_e - T_l)}{\sum_j m_j v_j^2} \qquad (4)$$

The sum goes over all atoms (atomic mass m) in the finite difference cell volume. Each MD timestep contains at least two finite difference timesteps. To stabilize the numerical solver, initially MD timestep of only $\Delta t = 2\times10^{-6}$ fs is used that gradually grows to $\Delta t = 0.1$ fs during the first picosecond.

The parameters of the heat equation are based on an electronic temperature dependent electronic heat capacity that is determined up to 10^5 K using *Quantum Espresso* [9,10]. For these computations, the diamond unit cell is relaxed, and the density of states, $g(E)$, is determined using a 16 × 16 × 16 Monkhorst-Pack k-point grid with kinetic energy cutoff of 200 Ry and a total of 100 bands. Computations are performed with the Projector-Augmented wave (PAW) technique and the Perdew-Burke-Ernzerhof functional (C.pbe-n-kjpaw_psl.1.0.0.UPF from *www.quantum-espresso.org*). From $g(E)$, the chemical potential is solved using the

bisection method and conservation of total number of electrons. Once the chemical potential is known, the heat capacity is determined by evaluating

$$C_e(T_e) = \int_{-\infty}^{\infty} \frac{\partial f(\mu(T_e),T_e)}{\partial T_e} g(E)EdE\ . \quad (5)$$

The effective electron-phonon coupling can be approximated as $G(T_e) = C(T_e)/\tau$, where τ is the exponential relaxation rate of the electronic-temperature without spatial gradients. Sadasivam et al. [11] has studied the electron-phonon relaxation based on the semiclassical transport equations using electron-phonon scattering rates as determined by density functional perturbation theory. The relaxation rate is determined by fitting a value of $\tau = 60$ fs to the electronic temperature evolution of diamond under uniform excitation (given in the Supplemental Material of Ref. [11], p. 24). The diffusivity value is not well known. We use a value of D_e =1.3 cm^2 s^{-1} to be consistent with earlier studies of SHI effects in nitrogen doped diamonds [12]. For 1 GeV Au ion (as used in Ref. [12]), continuous tracks appear below D_e = 1.0 cm^2 s^{-1} and there is almost no effect above D_e = 2.0 cm^2 s^{-1}. We have also tested that value up to D_e = 1.6 cm^2 s^{-1} gave similar results to those reported here.

$A(r_\perp)$ is the initial temperature determined by first estimating the initial energy density near the ion trajectory using the delta-ray production formula developed by Chunxiang [13], normalized so that it yields the electronic stopping power as predicted by SRIM 2010 code [14], and translating it to initial temperature by integrating the electronic heat capacity.

The simulation cells are prepared with a relaxation run using the Berendsen thermo- and baro-stat [15] to 300 K, 0 GPa for 50 ps. The initial energy deposition is calculated so that ion passes through the cell in the shortest direction (i.e., 13 nm), and the atoms near the four remaining outer boundaries are cooled to 300 K with a rapid Berendsen thermostat. To simulate the impact, the system is let to evolve for 100 ps. After this time, the resulting track radii are measured visually using at least four different locations at different sides of the track.

Supplementary Note 6. The highest electronic stopping power induced by monoatomic ions in diamond

Supplementary Figure 6 shows the energy dependence of the electronic stopping power S_e in diamond induced by three different ion species of Cu, Xe, and U. While S_e increases proportional to $E^{1/2}$ in the low energy region, it decreases in the high energy region, leaving a maximum which is called Bragg peak. The Bragg peak is higher for ions with higher atomic number Z. Consequently, the S_e value at the peak of the Bragg peak of U ion is practically the highest value attained by any monoatomic ions. If you want to know whether ion tracks are formed in a certain material by monoatomic ions, you can check whether ion tracks are formed at the peak S_e value induced by U ions.

GSI group reported that ion tracks have never been formed in diamond by GeV U ion irradiation [16] , whose maximum S_e reaches to 49.3 keV nm^{-1}. According to **Figure 3d in the**

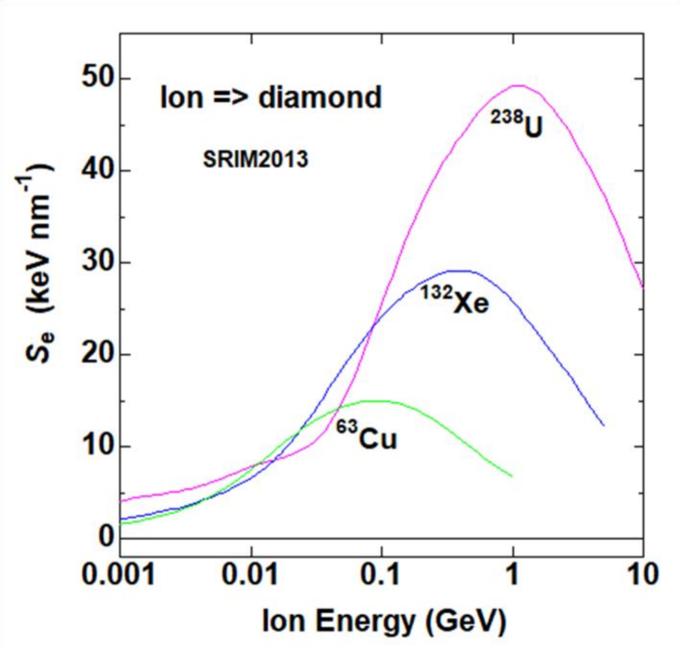

Supplementary Figure 6. Energy dependence of electronic stopping power S_e for three ion species, Cu, Xe, and U, injected into diamond, calculated by SRIM 2013 code. Each ion shows the Bragg peak, i.e., the S_e maximum. The maximum S_e value increases with the atomic number Z of the incident ion.

main text, the tracks have been observed between 2 MeV and 9 MeV C_{60} ion irradiations, which correspond to S_e between 29 and 52 keV nm^{-1}. As shown in **Supplementary Figure 6**, similar values of S_e are provided from U ions in the GeV region.

Supplementary Note 7. Almost one grain structures of microscopically thinned samples for TEM observation

Supplementary Figure 7 shows a bright field transmission electron microscopy (BF-TEM) image of a diamond sample in the low-magnification mode, which was thinned down for the TEM observation. The observations were carried out at thinner half, i.e., the right half in this case, of the sample. While the original diamond samples were macroscopically polycrystalline, the TEM observations showed that microscopic samples, which were thinned down for TEM observations, consist of almost one grain. During the ion irradiation and the TEM observation, the samples can be regarded as single crystals.

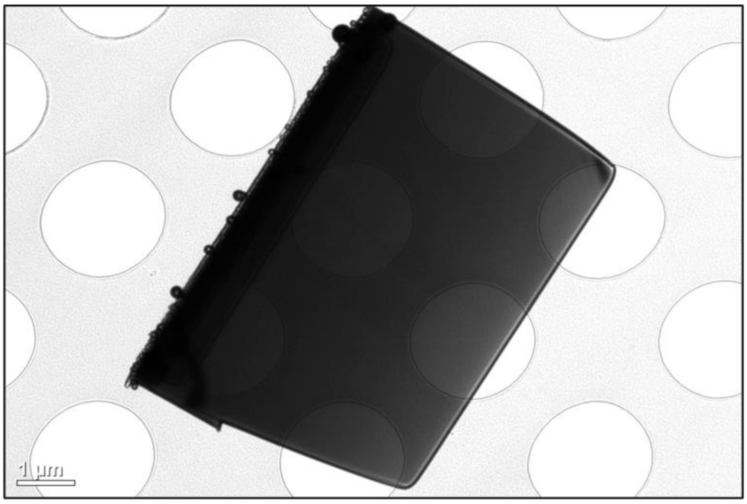

Supplementary Figure 7. A BF-TEM image of a diamond sample in the low magnification mode. No grain boundary was observed, i.e., the TEM samples are regarded as a nearly single crystal.

Supplementary Note 8. Purity of C_{60} cluster ion beam

Samples were irradiated with C_{60} ions of 1, 2, 4, 6, and 9 MeV using a tandem accelerator with the maximum terminal voltage of 3 MV. While the ions of 1-6 MeV were accelerated in the +1 charge state, i.e., C_{60}^{+}, only the ions of 9 MeV were accelerated in the +2 charge state, i.e., C_{60}^{2+}. Consequently, the +1 state ions, C_{60}^{+}, are well separated from fragments from C_{60} ions by analyzing magnet. However, C_{60}^{2+} ions are not free from the contamination of C_{30}^{+} ions, which are formed by the fragmentation of C_{60} ions, because both the C_{60}^{2+} and C_{30}^{+} ions have the same m/q ratio. **Supplementary Figure 8** shows experimentally detected intensities of C_{60}^{2+} beam (a peak around 850 ch) and of C_{30}^{+} beam (a peak around 580 ch) by the SSD. The C_{30}^{+} beam is much lower intense than C_{60}^{2+} beam and almost negligible.

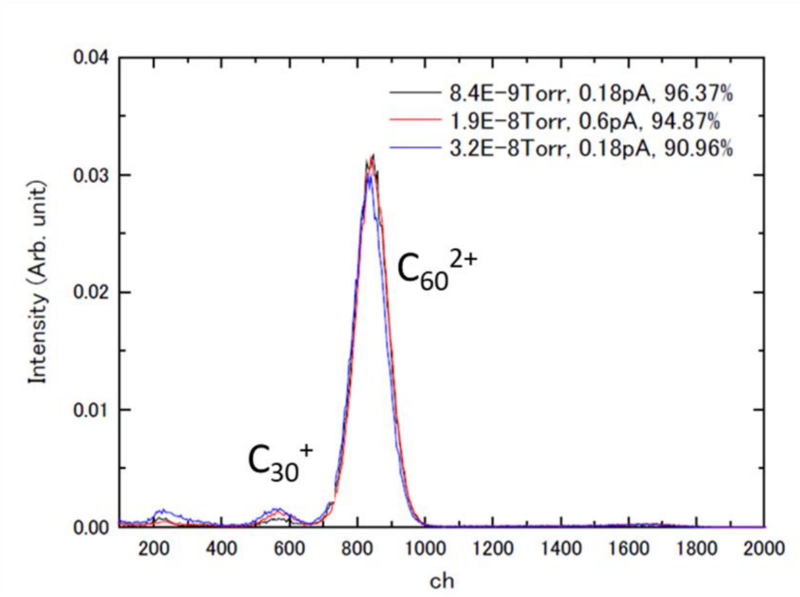

Supplementary Figure 8. Beam intensity of C_{60}^{2+} ions for 9 MeV irradiation against the C_{30}^{+} ions, detected by an SSD. Since C_{60}^{2+} and C_{30}^{+} ions have the same *m*/*q* ratio, they are not separated by analyzing magnet. However, the C_{30}^{+} intensity was much lower than the C_{60}^{2+} intensity.

Supplementary Note 9. Validity of the independent sixty C ion model for estimating the stopping power of C_{60} ion

The electronic and nuclear stopping power S_e and S_n of C_{60} ions are approximated from following relationship:

$$S_i(E, C_{60}) = \gamma_i N \cdot S_i(E / N, C_1) \tag{6}$$

where $i = e$ (electronic) [17] or $i = n$ (nuclear) [18], and $N = 60$ is presumed for C_{60} ions. The stopping powers of the monoatomic ions $S_i(E, C_1)$ were calculated with SRIM 2013 [19]. The coefficient $\gamma_i = 1$ is assumed in eq. (6), while it is rigorously valid only when the interaction between each C constituent is negligible. This could not be the case for the C_{60} ion, where sixty C atoms are injected simultaneously within the size of the C_{60} molecule, i.e., a diameter of 0.7 nm. A strong enhancement or reduction in the stopping was expected. Recently, Kaneko, however, excluded these possibilities on S_e by calculations in the energy range between 2 MeV and 10 MeV. He calculated the electronic stopping power of a C_{60} ion in an electron gas

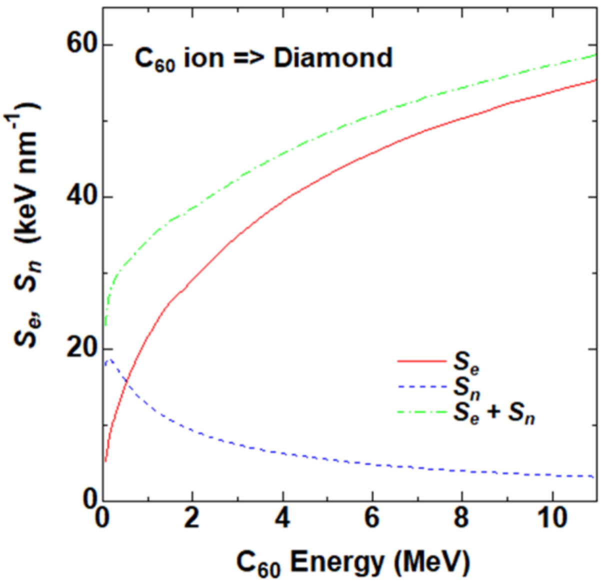

Supplementary Figure 9. Ion energy dependences of electronic and nuclear stopping power S_e and S_n of C_{60} ion injected to diamond, which were calculated from eq. (6).

corresponding to C foil, using the dielectric function theory with assuming that the shape and the size of the C_{60} molecule did not change [20]. He derived the value of γ_e, which weakly depends on the ion energy but is approximated as a constant of ~0.8 between 2 and 10 MeV. In this paper, $\gamma_1 = 1$ is assumed for eq. (6). **Supplementary Figure 9** shows the ion energy dependence of S_e and S_n of C_{60} ion injected in diamond, calculated from eq. (6).

Supplementary Methods 1: Two configurations of TEM sample fabrication (Top-view/ Side-view)

Two different configurations of TEM samples were prepared, namely (a) top-view configuration and (b) side-view configuration, as schematically depicted in **Supplementary Figure10.** Hereafter they are called briefly, e.g., top-view configuration or top-view sample. In the top-view configuration, TEM samples which are thin enough for the electron beam transmission are fabricated with focused ion beam (FIB) milling. Then the TEM samples were irradiated with C_{60} ions, and the top-views of the tracks are observed by TEM. In the side-view configuration, a bulk sample is firstly irradiated with the C_{60} ions. Then the cross-sectional samples are fabricated by the FIB milling.

However, since the diamond samples are very hard, further care was necessary. Basically, the sample heads, i.e., the parts face to the FIB, are protected by deposited carbon films. However, very huge FIB fluences are necessary for thinning of diamond. The sample heads are easily lost. An important trick is to rotate the sample at 90° and to irradiate one of the sides to the FIB. While a side of the surface region close to the beam is completely is lost, the other side survives almost without loss.

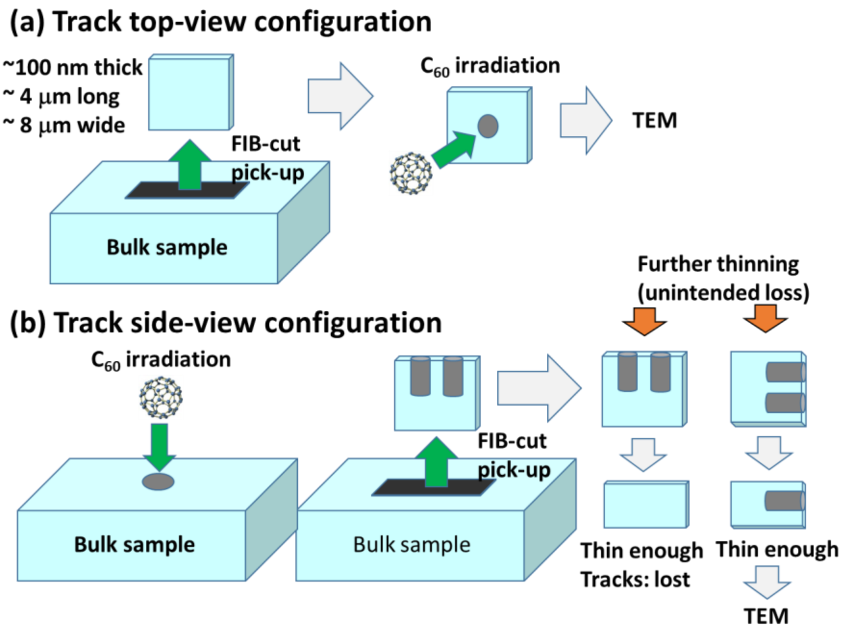

Supplementary Figure 10. Schematically depicted two configurations of TEM sample fabrication: (a) track top-view configuration and (b) track side-view configuration. Reproduced with permission from [21], ©2021 The authors.

Supplementary References

[1] Yasuda, K., Yamamoto, T., Etoh, M., Kawasoe, S., Matsumura, S. & Ishikawa, N. Accumulation of radiation damage and disordering in $MgAl_2O_4$ under swift heavy ion irradiation. *International Journal of Materials Research* **102**, 1082-1088; (2011).

[2] Jensen, J., Dunlop, A. & Della-Negra, S. Microscopic observations of metallic inclusions generated along the path of MeV clusters in CaF_2. *Nuclear Instruments and Methods in Physics Research Section B: Beam Interactions with Materials and Atoms* **146**, 399-404; (1998).

[3] Ivanov, D. S. & Zhigilei, L. V. Combined atomistic-continuum modeling of short-pulse laser melting and disintegration of metal films. *Physical Review B* **68**, 064114; (2003).

[4] K. Nordlund, PARCAS computer code. The main principles of the molecular dynamics algorithms are presented in [10, 11].

[5] Nordlund, K., Ghaly, M., Averback, R. S., Caturla, M., Diaz de la Rubia, T. & Tarus, J. Defect production in collision cascades in elemental semiconductors and fcc metals. *Physical Review B* **57**, 7556; (1998).

[6] Ghaly, M., Nordlund, K. & Averback, R. S. Molecular dynamics investigations of surface damage produced by kiloelectronvolt self-bombardment of solids. *Philosophical Magazine A* **79**, 795-820; (1999).

[7] Leino, A. A., Daraszewicz, S. L., Pakarinen, O. H., Nordlund, K. & Djurabekova, F. Atomistic

two-temperature modelling of ion track formation in silicon dioxide. *EPL (Europhysics Letters)* **110**, 16004; (2015).

[8] Tersoff, J. Modeling solid-state chemistry: Interatomic potentials for multicomponent systems. *Physical Review B* **39**, 5566-5568; (1989).

[9] Giannozzi, P. *et al.* QUANTUM ESPRESSO: a modular and open-source software project for quantum simulations of materials. *Journal of Physics: Condensed Matter* **21**, 395502; (2009).

[10] Giannozzi, P. *et al.* Advanced capabilities for materials modelling with Quantum ESPRESSO. *Journal of Physics: Condensed Matter* **29**, 465901; (2017).

[11] Sadasivam, S., Chan, M. K. Y. & Darancet, P. Theory of Thermal Relaxation of Electrons in Semiconductors. *Phys. Rev. Lett.* **119**, 136602; (2017).

[12] Lake, R. E., Persaud, A., Christian, C., Barnard, E. S., Chan, E. M., Bettiol, A. A., Tomut, M., Trautmann, C. & Schenkel, T. Direct formation of nitrogen-vacancy centers in nitrogen doped diamond along the trajectories of swift heavy ions. *Appl. Phys. Lett.* **118**, 084002; (2021).

[13] Chunxiang, Z., Dunn, D. E. & Katz, R. Radial Distribution of Dose and Cross-Sections for the Inactivation of Dry Enzymes and Viruses. *Radiation Protection Dosimetry* **13**, 215-218; (1985).

[14] Ziegler, J. F., Ziegler, M. D. & Biersack, J. P. SRIM – The stopping and range of ions in matter (2010). *Nuclear Instruments and Methods in Physics Research Section B: Beam Interactions with Materials and Atoms* **268**, 1818-1823; (2010).

[15] Berendsen, H. J. C., Postma, J. P. M., van Gunsteren, W. F., DiNola, A. & Haak, J. R. Molecular dynamics with coupling to an external bath. *The Journal of Chemical Physics* **81**, 3684-3690; (1984).

[16] Lang, M., Glasmacher, U. A., Neumann, R., Schardt, D., Trautmann, C. & Wagner, G. A. Energy loss of 50-GeV uranium ions in natural diamond. *Applied Physics A* **80**, 691-694; (2005).

[17] Ben-Hamu, D., Baer, A., Feldman, H., Levin, J., Heber, O., Amitay, Z., Vager, Z. & Zajfman, D. Energy loss of fast clusters through matter. *Phys. Rev. A* **56**, 4786-4794; (1997).

[18] Bouneau, S. *et al.* Very large gold and silver sputtering yields induced by keV to MeV energy Au_n clusters (n = 1–13). *Physical Review B* **65**, 144106; (2002).

[19] Ziegler, J. F., Biersack, J. P. & Ziegler, M. D. *SRIM - The Stopping and Range of Ions in Matter*. (SRIM Co., Chester, MD, 2008).

[20] Kaneko, T. MeV Cluster Ion Beam-Material Interaction. *Quantum Beam Science* **6**, 6; (2022).

[21] Amekura, H. *et al.* Ion tracks in silicon formed by much lower energy deposition than the track formation threshold. *Scientific Reports* **11**, 185; (2021).
